# Structural Insights into σ_1_ Receptor Interactions with Opioid Ligands by Molecular Dynamics Simulations

**DOI:** 10.3390/molecules23020456

**Published:** 2018-02-18

**Authors:** Mateusz Kurciński, Małgorzata Jarończyk, Piotr F. J. Lipiński, Jan Cz. Dobrowolski, Joanna Sadlej

**Affiliations:** 1 Faculty of Chemistry, University of Warsaw, Pasteur Str. 1, 02-093 Warsaw, Poland; 2 National Medicines Institute, 30/34 Chełmska Str., 00-725 Warsaw, Poland; m.jaronczyk@nil.gov.pl (M.J.); j.dobrowolski@nil.gov.pl (J.Cz.D.); 3 Department of Neuropeptides, Mossakowski Medical Research Center, Polish Academy of Sciences, 02-106 Warsaw, Poland; plipin@icm.edu.pl; 4 Faculty of Mathematics and Natural Sciences. Cardinal Stefan Wyszyński University, 1/3 Wóycickiego Str., 01-938 Warsaw, Poland

**Keywords:** sigma receptor, opioid ligands, molecular dynamics, fentanyl, morphine, haloperidol

## Abstract

Despite considerable advances over the past years in understanding the mechanisms of action and the role of the σ_1_ receptor, several questions regarding this receptor remain unanswered. This receptor has been identified as a useful target for the treatment of a diverse range of diseases, from various central nervous system disorders to cancer. The recently solved issue of the crystal structure of the σ_1_ receptor has made elucidating the structure–activity relationship feasible. The interaction of seven representative opioid ligands with the crystal structure of the σ_1_ receptor (PDB ID: 5HK1) was simulated for the first time using molecular dynamics (MD). Analysis of the MD trajectories has provided the receptor–ligand interaction fingerprints, combining information on the crucial receptor residues and frequency of the residue–ligand contacts. The contact frequencies and the contact maps suggest that for all studied ligands, the hydrophilic (hydrogen bonding) interactions with Glu172 are an important factor for the ligands’ affinities toward the σ_1_ receptor. However, the hydrophobic interactions with Tyr120, Val162, Leu105, and Ile124 also significantly contribute to the ligand–receptor interplay and, in particular, differentiate the action of the agonistic morphine from the antagonistic haloperidol.

## 1. Introduction

The growing number of crystal structures of the receptors has shed a strong light on their structural features, the arrangement of the transmembrane helices, ligand–binding interactions, and specificities. Very recently, two high-resolution crystal structures of the human transmembrane σ_1_ receptor in complex with two ligands (a high-affinity antagonist PD144418 and either agonist or inverse agonist 4-IBP; PDB ID: 5HK1 and 5HK2, respectively) have been published for the first time by Schmidt et al. [1]. The amino acid sequence of the σ_1_ receptor does not resemble any of the other mammalian proteins. To the best of our knowledge, up until now, no molecular dynamics (MD) simulations using the crystal σ_1_ receptor structure has been published.

**Figure 1:**
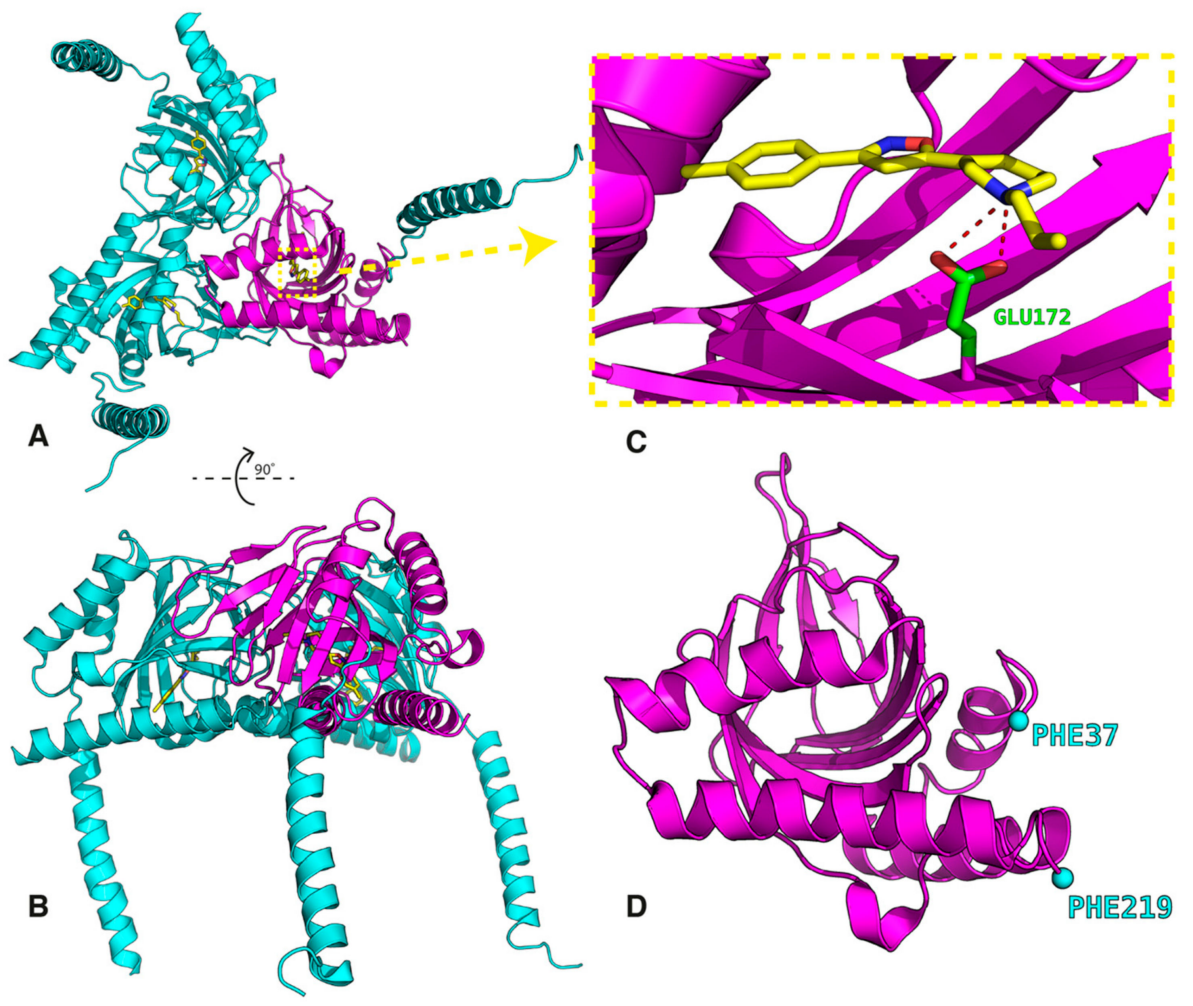
Structure of the human sigma 1 (σ_1_) receptor (the data were taken from file PDB ID: 5HK1 [1]). Complete trimer (**A** and **B**), the ligand PD144418 binding site (**C**), and the fragment of the receptor extracted for the docking simulations (**D**).

The crystal data show a triangular structure constituted of three associated units with a single transmembrane domain for each protomer (Figure 1) located at the N-terminus [1]. The three associated protomers, consisting of four α-helices each, form a flat triangle with a transmembrane domain at each corner. The receptor binding pocket is hydrophobic but the receptor–ligand interactions known from X-ray structures are formed through the ligand hydrogen bond with Glu172, which interacts with Tyr103 and Asp126 residues. The two compounds, antagonist PD144418 and agonist/inverse agonist 4-IBP, bind with very similar poses despite modest chemical similarity [1].

The sigma 1 (σ_1_) receptor is an endoplasmic reticulum integral membrane protein receptor. It is widely distributed in different areas of the central nervous system (CNS). Its ligands appear to be useful in different socially important therapeutic fields, such as treating depression, anxiety, amnesic and cognitive deficits, psychosis, and analgesia and in the treatment of drug abuse. In addition, the σ_1_ receptor plays a role in blocking the neurodegeneration caused by β-amyloid (Alzheimer’s disease) and in inhibiting tumor-cell proliferation and has potential in many other therapies [2,3,4,5,6,7,8,9]. This receptor has been characterized as a chaperone protein, which assists in the correct folding of some other proteins [7]. Also, potassium and calcium ion channels have been shown to be a target for the σ_1_ receptor ligands [9]. The receptor dynamically translocates itself inside cells and modulates intracellular Ca^2+^ signaling through the IP_3_ (inositol triphosphate) receptor [10].

The σ_1_ receptor binds a chemically diverse range of small or larger molecules as ligands. To date, the endogenous ligand of the σ_1_ receptor has yet to be identified as the σ_1_ receptor is unrelated to the aminergic G protein-coupled receptors (GPCRs) in sequence. However, given the close parallels in the binding site structure of the D2 dopamine receptor, the σ_1_ receptor has been postulated as an endogenous receptor that can modulate a number of neurotransmitter systems, such as the glutamatergic, noradrenergic, neurosteroid (in particular progesterone), and dopaminergic systems [7,8,9,10,11,12,13]. The pharmacological similarity between the σ_1_ receptor and other cholesterol-binding proteins led to the hypothesis on the cholesterol binding properties of this receptor (two regions: steroid binding domain-like (SBDL) I and II) [7,13].

Several exogenous ligands have been shown to bind with high affinity and selectivity to the σ_1_ receptor, although they are characterized by multiple structural dissimilarities [2,7]. The receptor exhibits affinity and selectivity for the (+)-benzomorphans (such as (+)-pentazocine or (+)N-allylnormetazocine SKF-10,047) [2], antipsychotics (such as haloperidol, antidepressants (fluoxetine, fluvoxamine, igmesine, imipramine, citalopram) [7,14,15,16,17,18,19,20,21], and neurosteroids (including progesterone (PGRMC-1 protein)) [7], as well as activity in processes that are important in cancer progression [6,7,15]. Also, there is evidence that the N,N-dimethyltryptamine (DMT), a well-known hallucinogen, is an endogenous σ_1_ receptor ligand [22].

The (+)-pentazocine was identified as a highly selective ligand for the σ_1_ receptor and was thus established as the primary selective radioligand for the determination of binding assays [2]. The experimental studies indicate that ligands have been classified as agonists or antagonists on the basis of their effects in animals. Whereas the benzomorphans are classified as agonists, ligands such as haloperidol are marked as antagonists [2,23,24].

Recently, a new synthetic class of σ_1_ receptor ligands have been obtained and studied using molecular dynamics (MD) simulations with a homology model of the receptor [25]. This class of receptor has been developed using as templates the 3D structures of four different proteins, structure–affinity relationships, pharmacophore models, and the mutagenesis data as used by Brune et al. [2,4], Laurini et al. [25,26,27,28,29,30,31,32,33,34], and other research groups [7]. These compounds are alkyl and arylcarboxamide derivatives [32], arylalkylamines [30], benzoxazolones, and dioxolane-based compounds [33]. The enantiopreference toward compounds was also studied [23,29,31]. 

Despite the existence of a lot of experimental and calculated data on drug interactions with the σ_1_ receptor, more information about the different ligands may need to be considered, particularly that the receptor’s crystallographic structures are bound to two different σ_1_ receptor ligands: one of which is a known antagonist and the other may be an agonist [1,7]. Nonetheless, the two ligand–protein structures are very similar. This could be partly because this receptor does not share a sequence homology with any other mammalian protein [7]. Moreover, this could also be because the recently published X-ray data for the σ_1_ receptor show “little similarity to previous computational models“ [1,5] used so far in homology-based studies [25,26,27,28,29,30,31,32,33,34]. Here we focus on the σ_1_ receptor for which crystal structural data are available for the first time. The question that needs to be addressed is how is the σ_1_ receptor binding site able to accommodate chemically diverse ligands?

The design and development of new potent and selective σ_1_ ligands are important for medicinal chemistry. The pharmacophoric requirement for the selective and optimal ligand binding to the σ_1_ receptor is the presence of the following: (i) the piperidine nitrogen atom, which matches the positive ionizable residue in the binding site, (ii) the amide oxygen atom, which is able to accept a hydrogen bond from a donor residue of the receptor, and (iii) the two aromatic rings, which can be engaged in hydrophobic interactions. The family of fentanyl derivatives satisfies these requirements and hence they could possibly have a significant binding affinity towards the σ_1_ receptor.

The site-directed mutagenesis assays and the crystal structure of σ_1_ show that Glu172 and Asp126 are essential for the ligand binding [1]. Glu172 is hydrogen bonded by the protonated ligand's amine group, while Asp126, instead of interacting with the ligand, forms an intramolecular hydrogen bond with Glu172. To figure out the structural details of ligand binding to the σ_1_ receptor, we chose seven ligands. Historically, the σ receptors were classified as opioid receptors; thus, some of the chosen ligands are simultaneously typical μ-opioid receptor ligands: fentanyl (FENT), morphine (MORPH), haloperidol (HALO), (*R,S*)-pentazocine (PTZ), and (*R,S*)-phenazocine (PHZ). There are two reasons for such a choice of ligands: (i) Firstly, most evidence to date has dealt with the therapeutic possibility of the σ_1_ receptor antagonists (i.e., haloperidol) for the treatment of neuropathic pain since opioid enhances the effectiveness of medical treatment [2,7]; (ii) Secondly, the σ_1_ receptor belongs now to a set of known experimental structures [1]; therefore, it is poised for a reevaluation of the interaction of these “old” ligands toward a putative binding partner.

In this paper, we present and discuss the results of the molecular dynamics simulations of the σ_1_ receptor–ligand system based on the σ_1_ receptor crystal structure [1]. The aim is to sketch the mechanism of inducing distinct responses of the σ_1_ receptor to the chosen seven ligands. The study includes the following:

(i) Carrying out independent MD simulations for the top-ranked poses of each ligand;
(ii) Examining the frequency of contacts and patterns between different ligand moieties and the receptor; ligand and different receptor residues; maps of different ligand moieties vs. different receptor residues;
(ii) Determining the binding energies (ΔG_bind_) and decomposing them according to the molecular mechanics/Poisson–Boltzmann surface area (MM/PBSA) approach to identify the most important contributions from polar and nonpolar contacts. Decomposition of ΔG_bind_ refers to terms such as ionic bond (or salt bridge) and hydrogen bond where the latter contains also attractive van der Waals and hydrophobic interactions essential for stabilizing the structure;
(iv) Searching for a relationship/trend between ΔG_bind_ and hydrophobicity/hydrophilicity of the residues through the linear regressions based on the studied ligands being treated as a training set.

## 2. Method

To investigate the interactions between the σ_1_ receptor and the ligands, an automatic, multi-stage protocol was designed.

### 2.1. Ligand Preparation

**Scheme 1:**
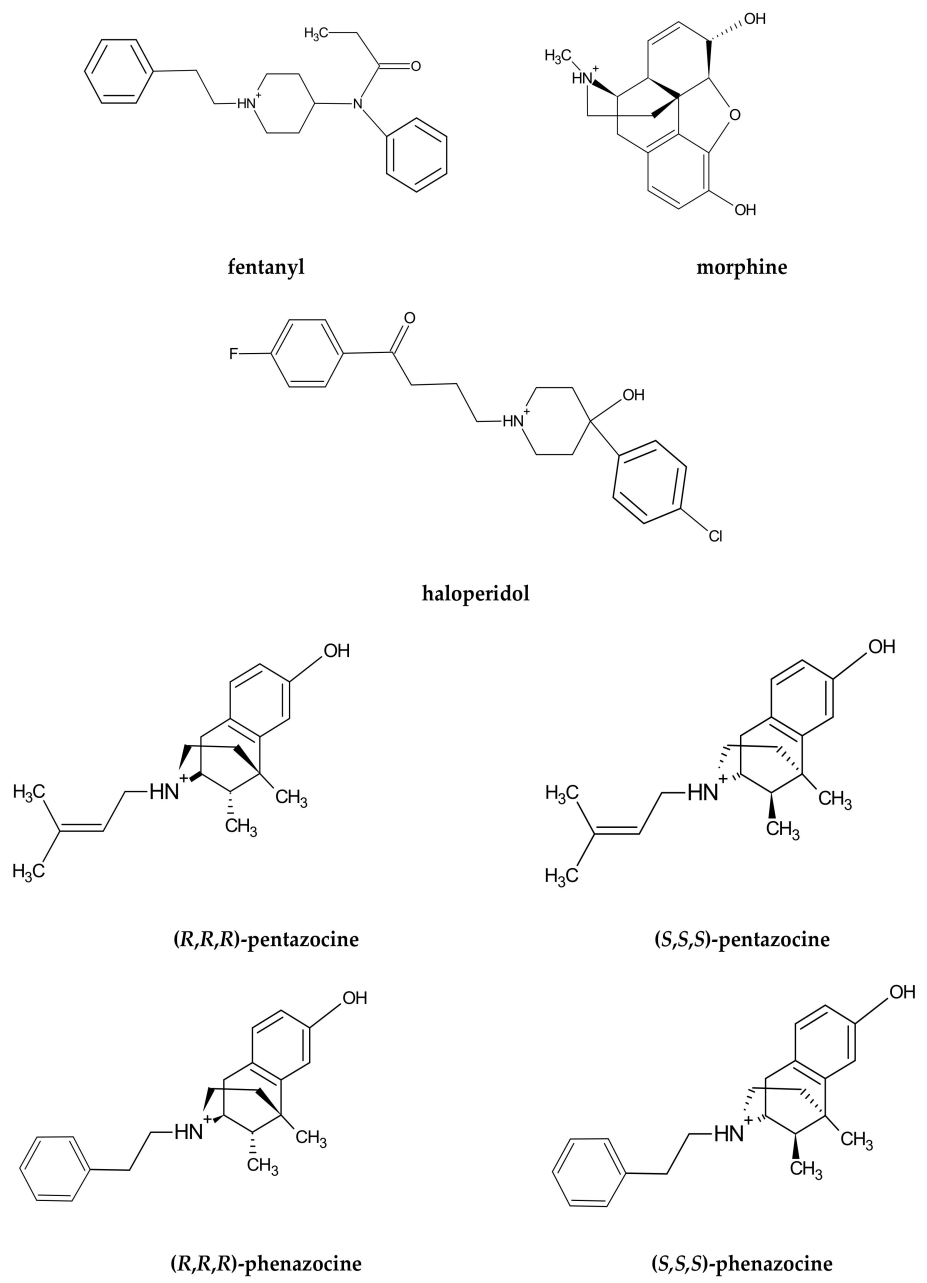
Structural formulae of the studied ligands.

The ligands (see Scheme 1) were prepared in the following fashion. First, sketch structures were drawn in the JSME applet. JSME is a 2D sketcher; however, it allows the drawing of bonds extending above and below the drawing plane. 2D sketches were automatically converted to naive 3D models, which took into consideration only atomic hybridization, and submitted to the Automated Topology Builder (ATB) [35]. The ATB server returned the optimized 3D structures and corresponding GROMACS [36] topologies. The retrieved files were used as input for the MD simulations.

### 2.2. Receptor Preparation

The receptor structure was taken from the 5HK1 entry of the PDB database [1]. This deposition contains the σ_1_ receptor homotrimer bound with three PD144418 ligand molecules, two lipid molecules, and sulfate ions. It is the higher resolution antagonist-bound crystal structure, one of two published by Schmidt et al. [1]. Chain B of the receptor was extracted only from the 5HK1 structure for further modeling. Additionally, the N-terminal helix (residues 1–36), which anchors the receptor in the membrane, was removed as it does not interact with either the ligand or its surroundings. The structure was refined using Modeller 9.18 [37] as a few residues were missing several atoms. The protein backbone was kept intact during this procedure. Figure 1 presents the σ_1_ receptor ligand complex.

### 2.3. Molecular Docking of Ligands to the σ_1_ Receptor

The ligands were docked in silico to the crystal structure of the σ_1_ receptor using the AutoDock program [38]. The binding pocket, which was manually selected with the ‘Grid Box’ tool from AutoDockTools, ran on the crystallographic structure 5HK1 with its native ligand PD144418. The procedure consisted of two steps: (1) setting up a minimal box to completely enclose the ligand and (2) extending the box size by approximately 50% in all directions. Docking was performed with the default AutoDock settings as its main purpose was to prepare the initial configuration for the MD simulations. Three docking runs yielded 8–10 docking poses each out of which up to three were selected for further processing [39]. The selection criterion was the presence of the hydrogen bond between the ligand's protonated NH^+^ moiety and the C=O group of the Glu172 receptor's residue. Such a bonding was observed in the crystallographic structure and is thought to be the key to the interaction with the σ_1_ receptor ligands.

### 2.4. Molecular Dynamics Simulation of the Receptor–Ligand Complex

In order to refine complex structures obtained from the docking procedure for each pose, we ran a fully flexible 80 ns MD simulation using the GROMACS program [36]. Production simulation stages were preceded by the energy minimization and the NVT and NPT equilibrations. Simulations were conducted with explicit solvent molecules at T = 300 K and δt = 2 fs time step.

### 2.5. The Formal Analysis of the MD Simulations and Presentation

The intention of the MD analysis was to find key similarities and differences in the ligand binding patterns. To this aim, 3000 structural snapshots, separated by 20 ps intervals, were extracted from the terminal with 60 ns on each trajectory. The interaction “fingerprint” was obtained from IChem analysis [40], providing both information on the occurrence and type of the interaction. This analysis returned to us the averaged contact maps depicting “fingerprint” patterns of the ligands binding to the receptor. We kept the default IChem parameters [40]. The receptor response to the ligand was monitored by the root mean square deviations (RMSD) and root mean square fluctuations (RMSF), which were calculated based on the binding site residues (Figure S1).

### 2.6. MM/PBSA Calculations for Binding Free Energy

To evaluate the strength of the interactions between the ligand and the σ_1_ receptor, the binding energy (ΔG_bind_) was calculated within the framework of the molecular mechanics/ Poisson-Boltzmann surface area (MM/PBSA) calculation approach [41]. It is a validated methodology combining the molecular mechanics energy and implicit solvation models. The enthalpic contribution to the free energy reflects the strength and specificity of the interactions between both partners, and includes ionic, electrostatic, hydrogen and halogen bonds, weak C-H…π hydrogen bond and the interactions with the prevailing dispersion components: π-π stacking, van der Waals and hydrophobic interactions [42,43]. Our main aim has been to compare the differences in the ligands’ binding to the σ_1_ receptor; therefore, we did not include the calculation of the entropic terms ΔS and refer only to the relative binding energies rather than to the absolute binding energies [44]. Moreover, as the binding energy is highly sensitive to the input parameters, the MM/PBSA model is more relevant in only calculating the relative differences. In order to base only on statistically converged results, independent short MD simulations starting with initial different velocities rather than long single simulations were run. 

The components of the electrostatic ΔE_elect_, the van der Waals ΔE_vdW_, the polar ΔE_polarsol_ and the nonpolar ΔE_nonpolarsol_ calculated as SASA [45] will be discussed in Section 3.3. The decomposition of the interaction energies on a per-atom basis was also calculated. All these components were averaged over a series of snapshots from the corresponding trajectories. From the terminal 60 ns MD trajectory, we extracted 100 snapshots every 0.6 ns from the production trajectories and ran the MM/PBSA protocol on them using the GROMACS package [45]. 

### 2.7. Simulation of the PD144418 Ligand

In order to confront our methodology with experimental data, we performed the docking procedure on the PD144418 ligand that we had extracted previously from the 5HK1 PDB crystallographic structure. Our main goal with this task was to check if the ligand known to form a stable complex with the σ_1_ receptor would reside within the pocket throughout the simulation while maintaining crucial interactions. The pre-simulation procedure was exactly the same as for the previously examined seven ligands. The simulation was performed under the same conditions as before but for a longer time (100 ns). The RMSD between the best-docked pose and the crystallographic structure was 0.59 Å and the crucial interaction of Glu172 with the protonated nitrogen atom in the ligand was retained in 99% of the trajectory snapshots. These all lead us to the conclusion that our procedure is valid for providing atom-level insight into the interaction of the σ_1_ receptor with fentanyl ligands.

## 3. Results and Discussions

### 3.1. Dynamical Properties of the σ_1_ Receptor–Ligand Interactions 

The binding pocket includes polar (p) and hydrophobic (h) residues: Asp126(p), Trp89(p), Met93(h), Leu95(h), Leu105(h), Leu182(h), Phe107(h), Ile124(h), Trp164(p), Tyr103(p), Ser117(p), His116(p). Actually, the binding pocket is quite hydrophobic and its interior is occluded from the solvent [1]. The path of the ligand entry, as well as its exit, remains unknown [1]. It seems most likely that the ligands enter into the binding pocket in dynamic fluctuations of the system.

**Figure 2:**
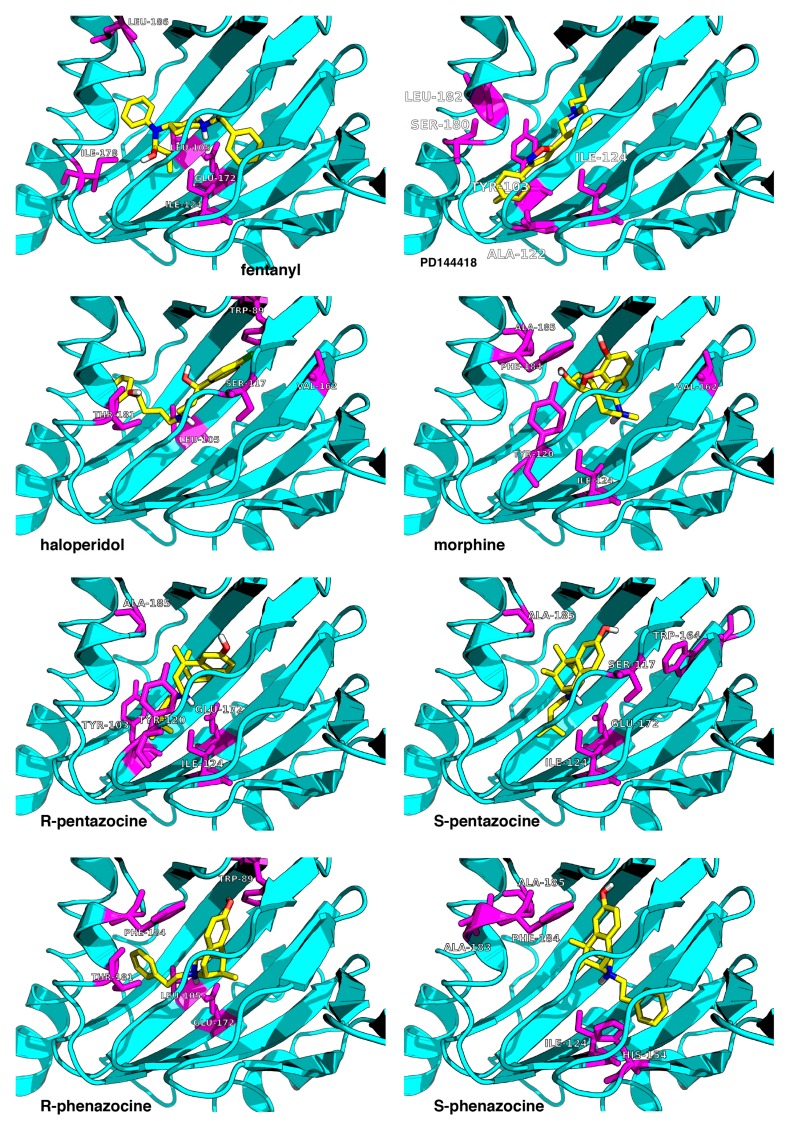
Top-scored docking poses for the seven studied ligands (yellow) bound to the receptor (cyan). The eighth ligand is PD144418 (top right corner). The receptor’s residues most frequently contacted during the MD simulations are shown in magenta. The image is a zoomed view of the receptor binding site.

Throughout this paragraph, we focus on the direct contacts between the ligand functional groups and the residues. Figure 2 illustrates the final snapshots of the MD simulations obtained for the seven ligands. Key interactions in the final snapshots are described below. First of all, the intramolecular salt bridge with the COO^−^ oxygen atom of Glu172 is present in all systems close to Asp126. In general, one can distinguish three groups of residues in the binding pocket:

(i) The first group is localized around Asp126 and forms the intermolecular salt bridge between the protonated nitrogen atom (N-H^+^) in the ligands and the carboxyl (COO^−^) group of the receptor Glu172 residue. We include also the stable hydrogen bonds between the carboxyl C=O group of Thr181 with the OH groups of the ligands in this region.
(ii) The second region is placed around the Tyr103 residue and plays a determining role in the π-π stacking interactions between Tyr103 and the aromatic/heteroaromatic rings of FENT, HALO, MORPH, PTZ, and PHZ.
(iii) It is noticeable that the residues (Phe107, Trp164, Ile178, Val162, Leu105, Leu182, and Ala185) from the third group belong to the hydrophobic pocket. They yield the required van der Waals and hydrophobic interactions, arranging appropriately the hydrophobic moieties of the ligands.

A more detailed description of the ligand–receptor interaction can be found in Table S1.

The conformational variations of the receptor upon the complex formation are presented in terms of the RMSD of the atoms’ positions with respect to their initial positions (Figure S1). The plots show that after about 20 ns of an initial increase of fluctuations, the receptor reaches equilibrium. The binding modes of the σ_1_ receptor–ligand are presented in Figure S2.

### 3.2. Ligand–Receptor Binding in Terms of the Frequency of Ligand–Receptor Contacts

The ligand–receptor interactions can be presented in a more quantitative way using the patterns of the frequency of contacts between:

(i) the ligand moieties with the receptor;
(ii) the receptor residues with the whole ligand;
(iii) the ligand–residue contact maps averaged over the MD trajectory.

**Figure 3:**
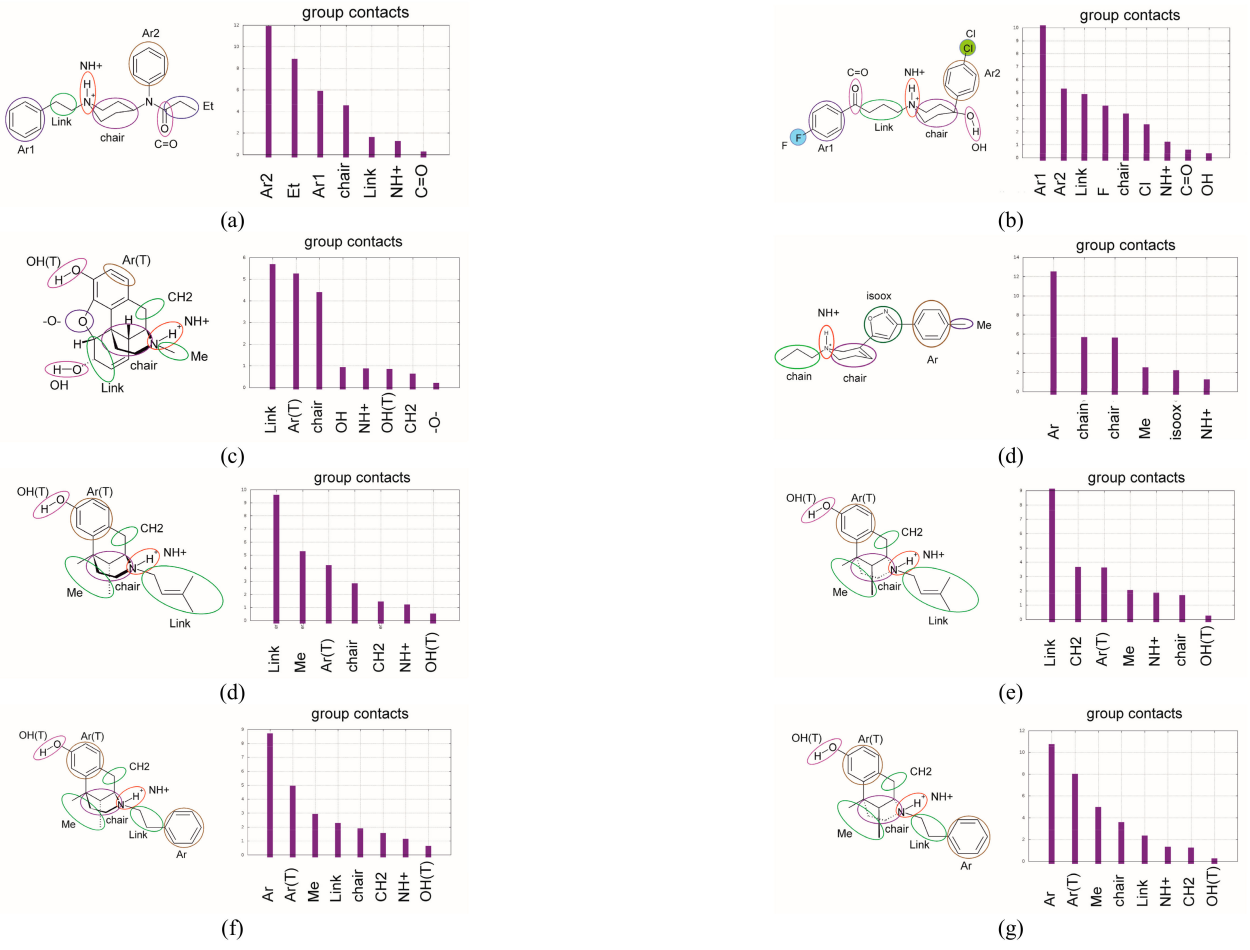
The contact analysis for the ligand moieties (left panel) with receptor (right panel) presents time-averaged histograms and the frequency of contacts with the moieties of ligands: (**a**) FENT, (**b**) HALO, (**c**) MORPH, (PD-144418), (**d**) *R*-PTZ, (**e**)*S*-PTZ, (**f**) *R*-PHZ, (**g**) *S*-PHZ.

The initial step of analysis of the contacts consists of the choice of moieties in the ligands participating in the interaction with the receptor (for abbreviations of moieties in the ligands, see Figure 3).

**Figure 4:**
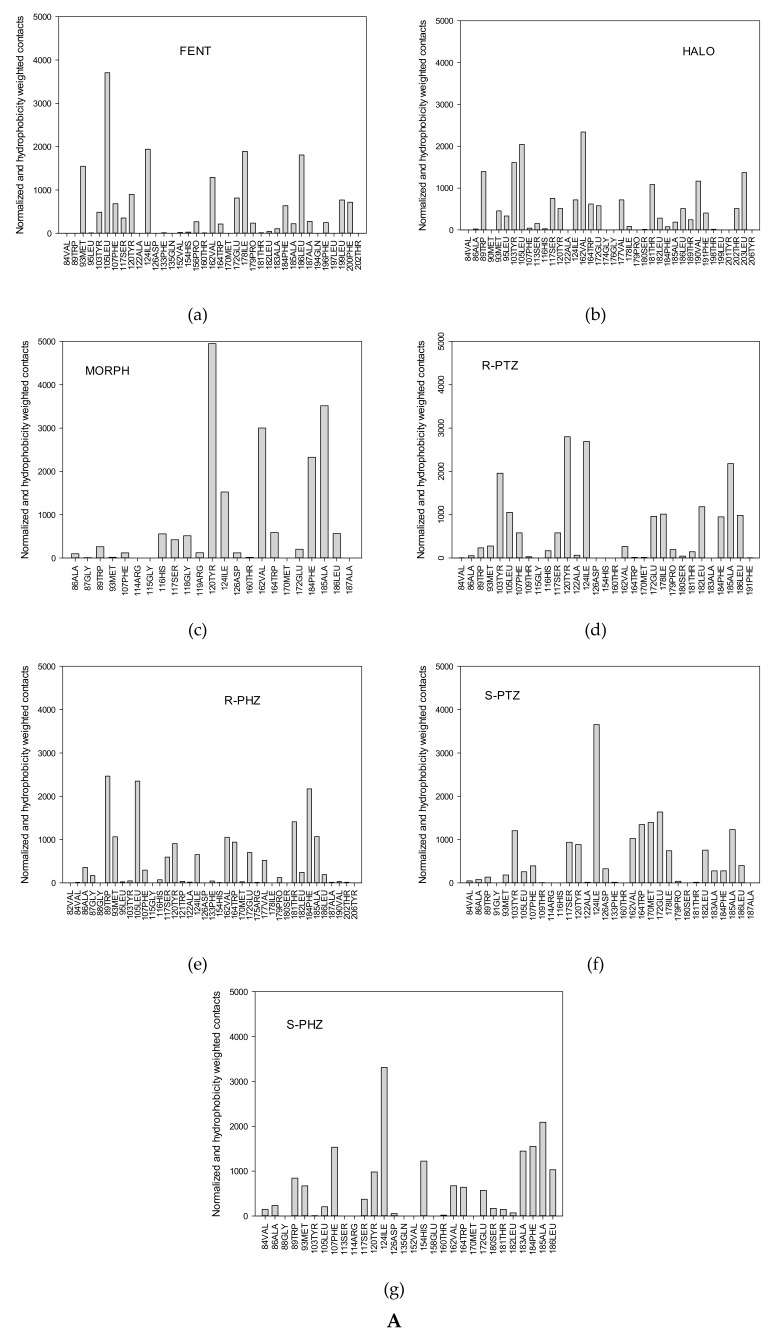

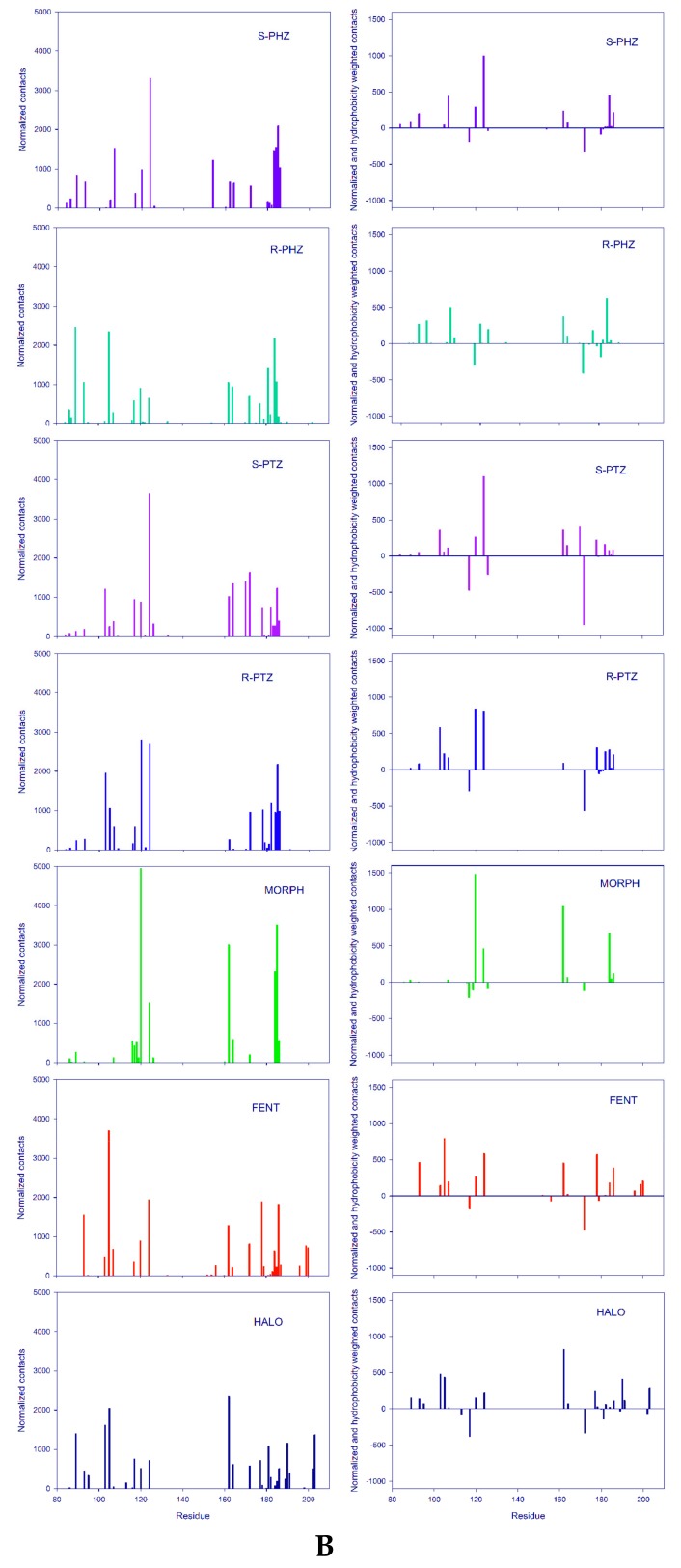
(**A**) (a–g) Normalized spectrum of the frequency of the contacts between receptor residues and the ligands; (**B**) On the left: Normalized spectrum of the frequency of the contacts between receptor residues and the ligands. On the right: Spectrum of the frequency of ligand contacts with receptor residues normalized according to the hydrophobic scale taken from Reference [46]. The positive bars correspond to hydrophobic contacts while negative bars correspond to hydrophilic contacts.

The search for the most frequently formed contacts led us to analyze the ligand–protein contact maps (Figure 3 and Figure 4). Figure 4A presents the normalized frequency of the receptor–ligand interactions, while Figure 4B on the right illustrates the frequency normalized by taking into account the hydrophobicity scale, published in [46]. The positive and negative signals correspond to the hydrophobic and hydrophilic residues, respectively. The first three ligands represent σ_1_ agonist (FENT), σ_1_ antagonist (HALO), and the ligand of reduced affinity to the σ_1_ receptor (MORPH). These ligands, apart from polar interactions (permanent salt bridge with the carboxylic group of the Asp126 and hydrogen bonds) are characterized by the π-π and hydrophobic contacts.

In the case of FENT, the most frequent contacts are with hydrophobic moieties (Ar2, Et, Ar1, chair, Link) while the contacts with hydrophilic moieties (NH^+^ and C=O) are rare (Figure 3a). In Figure 4Aa, the highest peaks are observed for five receptor residues: Leu105, Ile 124, Ile178, Leu186, and Glu172. Out of them, only Glu172 is hydrophilic. The other residues interact through the alkyl groups and significantly contribute to hydrophobic interactions. Several other residues that interact less frequently, such as Met93, Val162, and Leu199, additionally strengthen the hydrophobic interactions. Moreover, common interactions with Phe107, Phe184, and Phe200 introduce the π-π stacking interactions with the two fentanyl phenyl rings and increase the overall hydrophobic reception of this ligand. This interaction picture in which the dominant role is played by hydrophobic interactions that are strengthened by π-π stacking interactions and where hydrogen bonding plays a minor role would support the classification of fentanyl as a hydrophobic σ_1_ ligand. This is clearly supported by the relevant interaction spectra shown in Figure 3 and Figure 4A,B. Therefore, it may be attributed to the simplistic correlations between hydrophobicity and binding affinity. This allows us to perceive the importance of the hydrophobic character of amino acid chains seen in Figure 4.

In the case of HALO, the most frequent contacts are with the hydrophobic moieties (Ar1, Ar2, Link, F, chair, Cl) while the contacts with the hydrophilic moieties (NH^+^, C=O and OH) are less frequent (Figure 3b and Figure 4A,B). The highest peaks are observed in Figure 4A,B for the following receptor residues: Val162, Leu105, Tyr103, Trp89, Ser117, Val190, and Thr181. Notice that this time interactions with the hydrophobic (Leu, Val, and Leu) and hydrophilic (Trp, Thr, Ser, and Tyr) residues are found, although the hydrophilic ones are not as frequent and are of lower intensity than the hydrophobic ones. Observe also that in the case of HALO, more residues are active than for FENT (Figure 4A,B). Figure 3 illustrates the contact analysis for the co-crystallized ligand PD144418. Similar to HALO, the most frequent contacts are found with the hydrophobic moieties (Ar, chair).

A comparison between the frequency of interactions of the FENT and HALO moieties indicates that the hydrophobic moieties of both molecules are definitely more active than the hydrophilic ones. However, a comparison of the frequency of contacts with the σ_1_ receptor residues displays quite different patterns. For FENT, the majority of interactions occur with the hydrophobic residues. For HALO, contacts with the hydrophobic residues are frequent but the hydrophilic contacts are important as well (see Figure 4B). It may be important that only these two most strongly binding ligands exhibit interactions with residues with labels exceeding 190—that is, Phe196, Leu199, and Phe200 (FENT) and Leu199, Tyr201, Thr202, and Leu203 (HALO) (Figure 4B).

MORPH yields yet another interaction pattern. However, the hydrophobic moieties (Link, Ar(T) and chair) are responsible for the majority of molecular contacts (Figure 3c), while the hydrophilic residues Tyr120, Glu172, the hydrophobic Ile124 and Val162, and stacking forming Phe184 dominate in the frequency of contacts with the receptor. Therefore, the largest variety of different types of interactions are found in the case of MORPH, yet these interactions are focused on a much smaller number of residues (Figure 4A,B).

Four ligands, *R-* and *S*-PTZ plus *R*- and *S*-PHZ, confirm the experimental observations about the importance of the ligand's stereochemistry. According to Sguazzine et al. [47], the stereochemistry of the fenpropimorph is not important for the binding to the σ_1_ receptor, while the results found by Rossi et al. [29] suggest that in the case of the (*R*,*S*)-RC-33 ligands, there are preferences toward the *S*-conformation when the OH group is present in the chirality center. Hence, it is interesting how the PTZ and PHZ chirality affects the ligand–protein interactions.

In the case of *R*-PTZ, the most frequent contacts are with the hydrophobic moieties (Link, Me, Ar(T), chair, and CH_2_), while the contacts with the hydrophilic moieties (NH^+^ and OH(T)) are less frequent (Figure 3d). The highest peaks in Figure 4A are observed for the following residues: Tyr120, Ile124, Ala185, Tyr103, Leu182, Leu105, Ser117, and Glu172. Notice that this time the interactions with the hydrophobic (Leu, Ile, and Ala) and hydrophilic (Tyr, Glu, Ser, and Tyr) residues are, roughly, equally frequent. Interestingly, in agreement with the mutagenesis studies [4], frequent contacts of Tyr120 with *R*-PTZ can stabilize the ligand–receptor interaction and increase the potency of the *R*-form. A quite similar pattern is obtained for the *S*-PTZ (Figure 3f): the Link, CH_2_, Ar(T) and Me moieties form the most important hydrophobic interactions. However, two pairs of polar–hydrophobic contacts—Ile124 with Glu172 and Tyr120 with Ala185—seem to be promoting the leading contacts with high intensity suggesting the importance of the *S*-PTZ ionic interactions (Figure 4B).

In contrast to *R*-PTZ, the *R*-PHZ has hydrophilic contacts with Trp89, Leu105, Phe184, Glu172, Thr181, Trp164, and Tyr120 residues (Figure 4A), while the contacts with Leu105, Ile124, Phe184, and Ala185 residues have a hydrophobic character. However, the latter interactions are less frequent and play a minor role. In the case of *S*-PHZ (Figure 4A), the hydrophobic contacts with Ile124, Ala185, Phe184, Phe107, and Leu186 have definitively higher frequencies. Nevertheless, less frequent hydrophilic constants with His154 and Glu172 have also been noticed (Figure 4B). These findings suggest that *R*- and *S*-PHZ enantiomers display small but visible differences when binding to the σ_1_ receptor. In conclusion, the chiral preferences of the σ_1_ receptor seem to be clear yet they need further studies.

### 3.3. The Binding Energy Analysis

**Figure 5:**
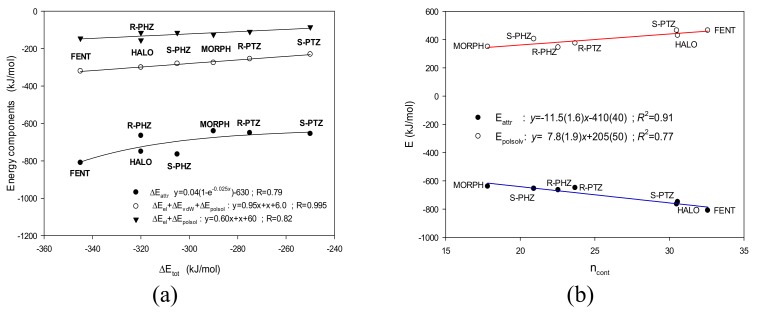
(**a**) Contribution of the most important energy terms to the total energy. (**b**) The attractive energy term ΔE_attr_ and the repulsive energy term ΔE_polarsol_ as a function of the number of contacts n_cont_ for the studied ligands.

The mutagenesis and crystallographic experiments revealed that the two polar, anionic σ_1_ residues Asp126 and Glu172 are obligatory for the ligand–receptor binding activity. In addition to those residues, several other hydrophobic residues (e.g., Ile128, Phe107, Lys105, Lys187, and Ala185) have been identified as playing a central role in the binding. Further insight into the origin of the ligand binding may be obtained from a decomposition of the total energy interactions (ΔE_tot_, the enthalpic contribution) into the electrostatic (ΔE_elect_), the van der Waals term, as well as the interactions with polar (ΔE_polarsol_) and non-polar solvents (ΔE_nonpolarsol_, within the SASA approximation). A comparison of the binding energy and its components for the series of the studied ligands within the MM/PBSA approach is presented in Table 1 and Figure 5.

First, it is noticeable that irrespective of the ligand’s structure, the relative contribution of (a) the electrostatic term to the total attractive energy is ca. 75%; (b) the attractive van der Waals energy is ca. 22%, and (c) the only repulsive term, the interactions with the polar solvent, is also somewhat constant and is equal to ca. 60%. For all ligands, the attractive electrostatic term overcomes the repulsive term and is the main driving force for the ligand–receptor binding (Table 1, Figure 5a). In general, the attractive terms and even the electrostatic term alone overcome the repulsive one. Thus, it seems that the absolute attractive interaction contributions play a crucial role rather than the differences in the relative contributions of the attractive and repulsive forces.

(A) Hence, the highest total binding energy is found for FENT, while the smallest is for *S*-PTZ. Moreover, even if the errors are taken into account and one considers only the low limits, the above conclusions remain true.
(B) On the other hand, the values of total interaction energies of the most strongly interacting ligands—FENT, HALO, *R*-PHZ, and *S*-PHZ—are within the error bars of each other. The same holds true for the most weakly interacting ligands—*S*-PTZ, *R*-PTZ, and MORPH—for which the total interaction energies are also within the error bars of each other.
(C) The high result for FENT is supported by both the highest electrostatic term and one of the highest van der Waals. FENT exhibits the largest attractive contribution and despite the fact that the repulsive term for it is also one of the two largest, the difference is still the biggest.

Notice that both the sum of the energy of the attractive interactions ΔE_attr_ and the repulsive energy term ΔE_polarsol_ linearly correlate with the number of contacts n_cont_ (see Figure 5b). Thus, those two relatively independent computational measures of the ligand binding affinity are in close alignment. 

Let us now juxtapose the results of the calculations with the experimental characteristics, bearing in mind that those two refer to quite different conditions. We can remark that:

(i) MORPH with the value of K_i_ > 10000 nM [11] is endowed with the lowest affinity toward the σ_1_ receptor among the series of studied compounds. This is consistent with both frequency of contacts and the most important terms of the interaction energy (Figure 5b).
(ii) Also, *S*-PHZ, which exhibits one of the highest K_i_ in the studied series, 61.7 nM [23], is characterized by almost the smallest number of contacts and the smallest interaction energy terms in the presented calculations (Figure 5b).
(iii) On the other hand, the HALO ligand, which is the strongest σ_1_ receptor antagonist considered here (K_i_ = 0.65 nM [13]) exhibits the second highest number of contacts and the attractive energy term.
(iv) Unfortunately, the computational predictions of the FENT binding energy were not successful. For fentanyl, the experimental K_i_ was estimated to be greater than 1000 nM [48,49], whereas in our calculations both the number of contacts and energy terms indicate FENT to interact very strongly (Figure 5b).
(v) The other studied ligands, *R*-PHZ, *R*-PTZ and *S*-PTZ, are located somewhere between the extreme interactions. However, they fit the predicted tendencies (Figure 5b).

At this moment we can state that there is in fact only one basic disagreement produced by the simulations with the FENT ligand. Although the experimental data can be thought to not be very exact, in the case of fentanyl the possibility of a mistake is very low. However, for now, we have no satisfactory explanation for the prediction of the very strong interaction between the FENT ligand and the σ_1_ receptor. In fact, several factors can be responsible for such a disagreement. Still, most of these factors would refer to all groups of ligands. Nevertheless, out of these factors, the number of FENT contacts with the Leu105 residue is definitely the highest in the group of studied ligands (Figure 4Aa). The next highest number of such contacts is for HALO. Therefore, these contacts are probably overestimated for fentanyl.

Nevertheless, we think that the qualitative characteristics of the ligand interactions with the hydrophilic and hydrophobic residues of the receptor can differentiate the studied ligands in a good way. Indeed, among the set of studied ligands, both HALO and FENT share the most hydrophobic interactions with the receptor residues and they are essential for ligand binding as an antagonist. Our simulations confirm the important role of the interactions with the hydrophobic (Leu, Val and Leu) and hydrophilic (Trp, Thr, Ser, Glu and Tyr) residues, which are also identified in the experiment structure as crucial for the binding process [1]. In contrast, MORPH is the ligand without frequent non-polar interactions, which disqualifies it from playing the role of an antagonist. Instead, we found more frequent interactions with polar residues for PTZ and PHZ than antagonists such as HALO. Therefore, the important polar and non-polar contacts can be related to the agonistic and non-antagonistic properties of the ligands, respectively.

## 4. Conclusions

An interesting and intriguing aspect of the σ_1_ receptor is that it can bind a wide spectrum of ligands of very different structural classes and diverse pharmacological applications, including, recently, a potential drug target for cancer therapy [7]. In contrast to the advances made for the family of G protein-coupled receptors (GPCRs) [50], the molecular mechanism governing the σ_1_ receptor function remains obscure despite the profound insight obtained recently from the crystal structures [1]. Aiming to improve the understanding of the ligand binding of this receptor, we have performed, for the first time, MD simulations for seven ligand–receptor complexes (fentanyl, haloperidol, morphine, (R,S)-pentazocine, (R,S)-phenazocine) and additionally for PD144418 ligand, based on the published crystal structure of the σ_1_ receptor (PDB ID: 5HK1). The combined approach (the MM/PBSA and the fingerprints analysis) unveiled the substantial role exerted by the polar and hydrophobic residue contacts in ligand binding so as to maintain the high affinity of the receptor. Ligand–protein interactions were categorized into two major types: hydrophilic and hydrophobic.

The results presented herein provide dynamic insight into the optimal binding of agonists and antagonists of the σ_1_ receptor in atomic detail through computational modeling approaches. The altered interactions of agonists and antagonists with the σ_1_ receptor have been known for a long time; however, the structural basis of the binding affinity was still elusive. The simulation results confirm the important role of particular protein residues—identified in the crystallographic σ_1_ receptor–ligand data—which are crucial for the binding process. Analysis of the observed intermolecular contact frequencies together with the contact maps indicates that the hydrophilic interactions with Glu172, retained by all of the studied ligands, are a contributing factor to the affinity of the σ_1_ receptor–ligand interaction. However, a high degree of contribution by hydrophobic interactions with Tyr120, Val162, Leu105, and Ile124 can clearly differentiate the studied agonist (MORPH) ligands from the antagonists (HALO). We anticipate that the results presented herein will pave the way for a deeper understanding of σ_1_ receptor–ligand interactions, especially the fentanyl derivatives that have been a recent threat to public health in the form of illegal drugs.

## Figures and Tables

**Table 1:** Binding energy and its components for ligand interaction with the σ_1_ receptor (kJ/mol, errors in parentheses). The ΔE_nonpolarsol_ term is calculated within the SASA approximation.

Ligand ∆E	FENT	HALO	MORPH	*R*-PTZ	*S*-PTZ	*R*-PHZ	*S*-PHZ
ΔE_tot_	−345 (35)	−320 (20)	−290 (20)	−275 (20)	−250 (25)	−320 (25)	−305 (25)
ΔE_vdW_	−175 (20)	−185 (15)	−150 (15)	−145 (15)	−145 (15)	−145 (15)	−165 (15)
ΔE_elect_	−610 (40)	−545 (20)	−475 (20)	−485 (25)	−490 (25)	−500 (50)	−580 (30)
ΔE_nonpolarsol_	−22 (1)	−22 (1)	−16 (1)	−18 (1)	−18 (1)	−18 (1)	−19 (1)
ΔE_polarsol_	465 (35)	430 (15)	350 (20)	375 (25)	405 (40)	345 (60)	465 (25)
ΔE_attr_	−810	−750	−640	−650	−655	−665	−765
ΔE_vdW_/ΔE_tot_(%)	51	58	52	53	58	45	54
ΔE_elect_/ΔE_tot_ (%)	177	170	164	176	196	156	190
−ΔE_polarsol_/ΔE_tot_ (%)	135	134	121	136	162	108	152
ΔE_vdW_/ΔE_attr_ (%)	22	24	23	22	22	22	22
ΔE_elect_/ΔE_attr_ (%)	76	73	74	75	75	75	76
−ΔE_polarsol_/ΔE_attr_ (%)	58	57	55	58	62	52	60

## Supplementary Materials

Supplementary Materials are available online at www.mdpi.com/xxx/s1.
