# Supplementary material for: Structural Insights into σ1 Receptor Interactions with Opioid Ligands by Molecular Dynamics Simulations
Source: Molecules. 2018 Feb 18;23(2):456. doi: 10.3390/molecules23020456 (PMC6017133; doi:10.3390/molecules23020456)
Supplement: Supplementary file 1 [file molecules-23-00456-s001.pdf]

Supplementary Materials
to
Structural insights into σ_1 receptor interactions with opioid ligands by molecular dynamics simulations

Mateusz Kurciński, *[1] Małgorzata Jarończyk,[2,] Piotr F. J. Lipiński,[3]
Jan Cz. Dobrowolski,[2] Joanna Sadlej *[2,4]

[1] *Faculty of Chemistry, University of Warsaw, Pasteur Str.1, 02-093 Warsaw, Poland*
[2] *National Medicines Institute, 30/34 Chełmska Str., 00-725 Warsaw, Poland*
[3] *Department of Neuropeptides, Mossakowski Medical Research Center, Polish Academy of Sciences, 02-106 Warsaw, Poland*
[4] *Faculty of Mathematics and Natural Sciences. Cardinal Stefan Wyszyński University,1/3 Wóycickiego Str., 01-938 Warsaw, Poland*

E-mails: mkurc@chem.uw.edu.pl, m.jaronczyk@nil.gov.pl, plipin@icm.edu.pl, j.dobrowolski@nil.gov.pl, j.sadlej@uksw.edu.pl

Fig. S1. The average RMSD of the ligand-receptor complexes: FENT, HALO, MORPH, *R*- and *S*-PTZ, *R*- and *S*-PHZ; x (psec), y(Å).

Fig. S2. 2D schematic representation of the interaction between the σ_1 receptor and the seven ligands: FENT, HALO, MORPH, *R*- and *S*-PTZ, *R*- and *S*-PHZ.

Figure S3. Spectrum of the frequency of the contacts between receptor residues and atomic centers of ligands.

Figure S4, a-g. Spectrum of frequency of ligand contacts with receptor residues normalized according to the hydrophobic scale. The positive bars correspond to hydrophobic while negative to hydrophilic contacts.

Table S5. The contacts between residues in receptor-ligand detected in the final MD snapshots. The names of different regions of the ligands: Ar1, Ar2, Ar(T), chair, Link, Me, -CH_2, see Fig.3.

Fig.S1. The average RMSD of the ligand-receptor complexes: FENT, HALO, MORPH, *R*- and *S*-PTZ, *R*- and *S*-PHZ; x (psec), y(Å).

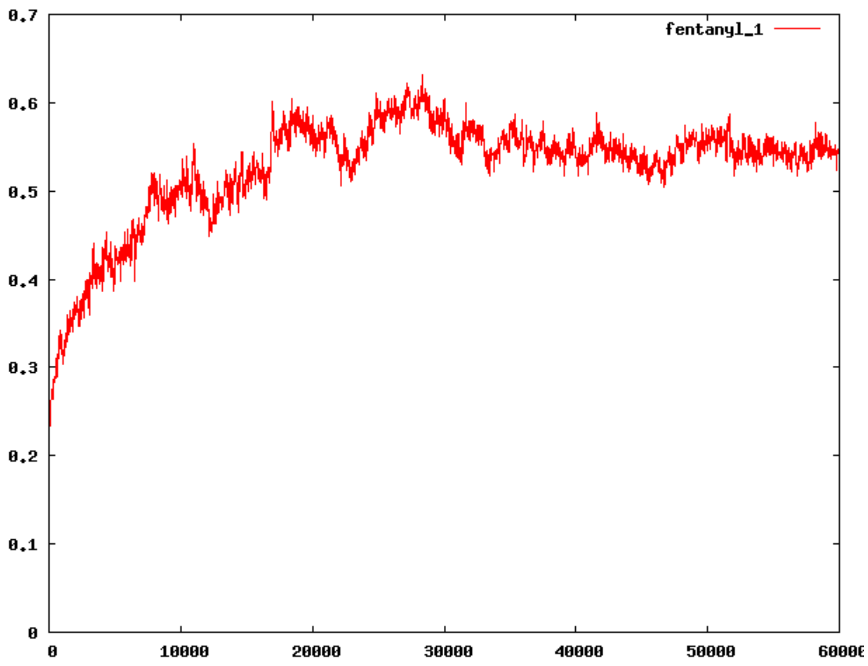

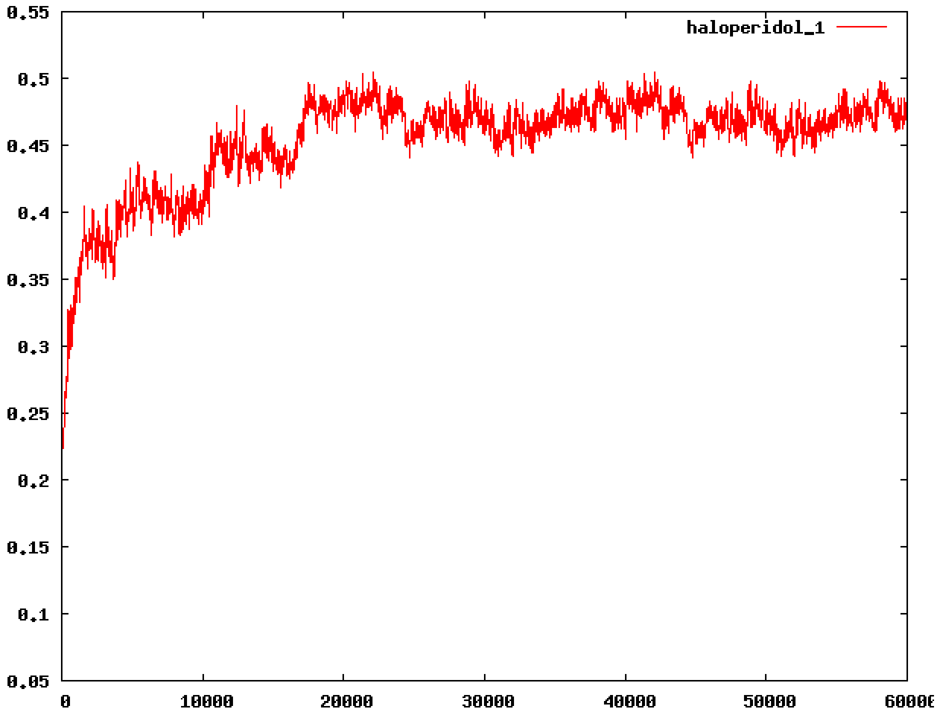

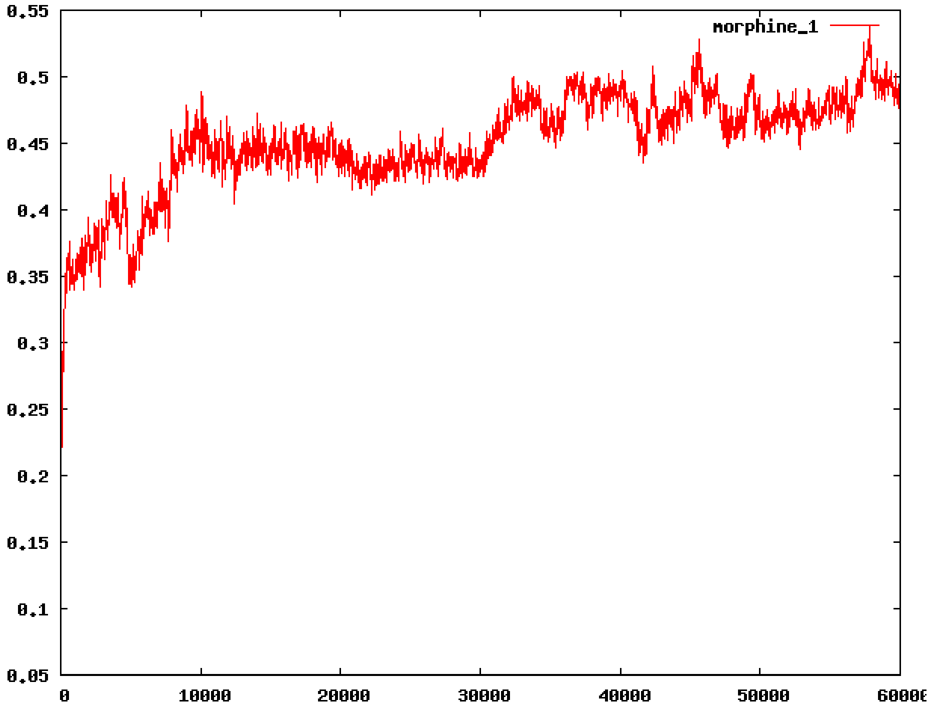
morphine_1
0.55
0.5
0.45
0.4
0.35
0.3
0.25
0.2
0.15
0.1
0.05
0
10000
20000
30000
40000
50000
60000

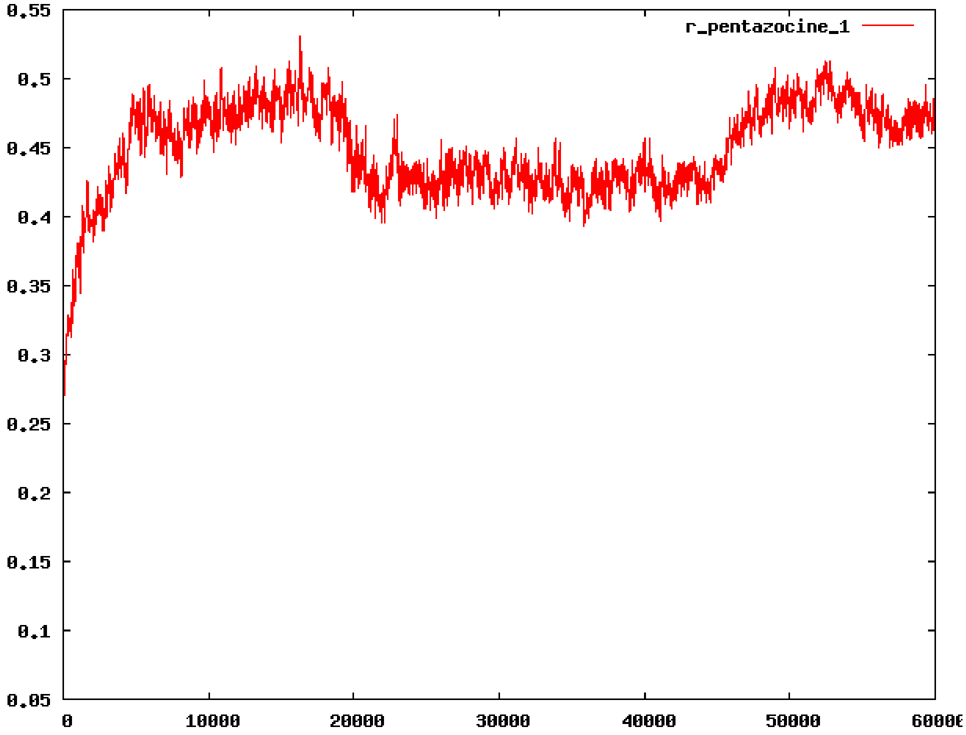
r_pentazocine_1
0.55
0.5
0.45
0.4
0.35
0.3
0.25
0.2
0.15
0.1
0.05
0
10000
20000
30000
40000
50000
60000

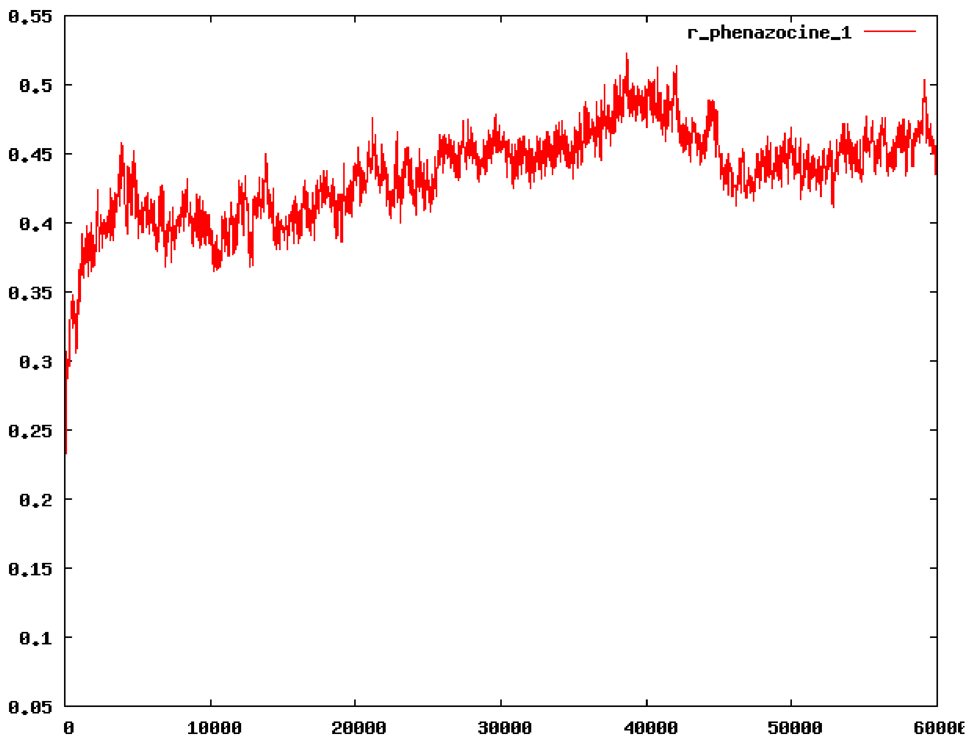
r_phenazocine_1
0.55
0.5
0.45
0.4
0.35
0.3
0.25
0.2
0.15
0.1
0.05
0
10000
20000
30000
40000
50000
60000

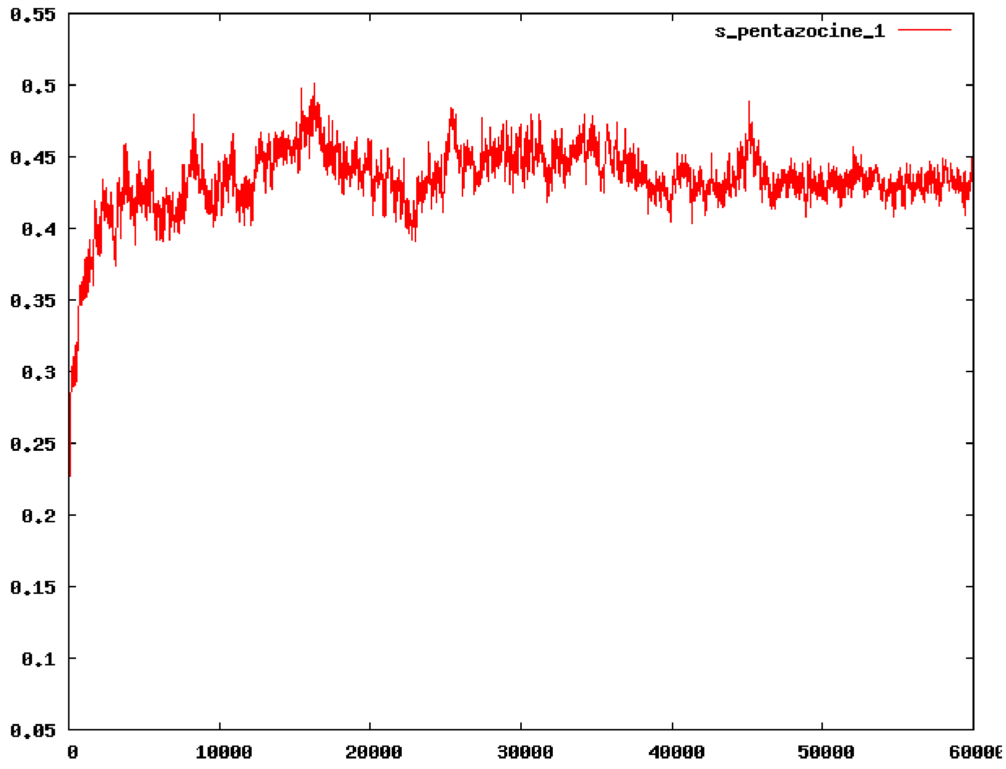
s_pentazocine_1
0.55
0.5
0.45
0.4
0.35
0.3
0.25
0.2
0.15
0.1
0.05
0
10000
20000
30000
40000
50000
60000

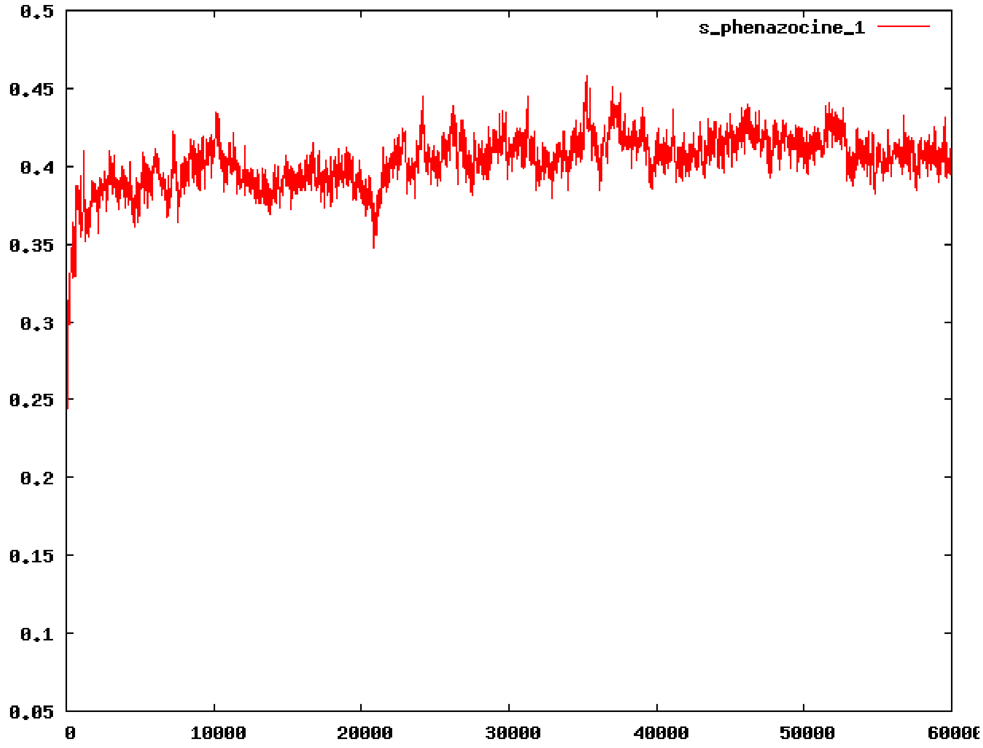

Fig.S2. 2D schematic representation of the interaction between the σ_1 receptor and the seven ligands: FENT, HALO, MORPH, *R*- and *S*-PTZ, *R*- and *S*-PHZ.

(a) FENT

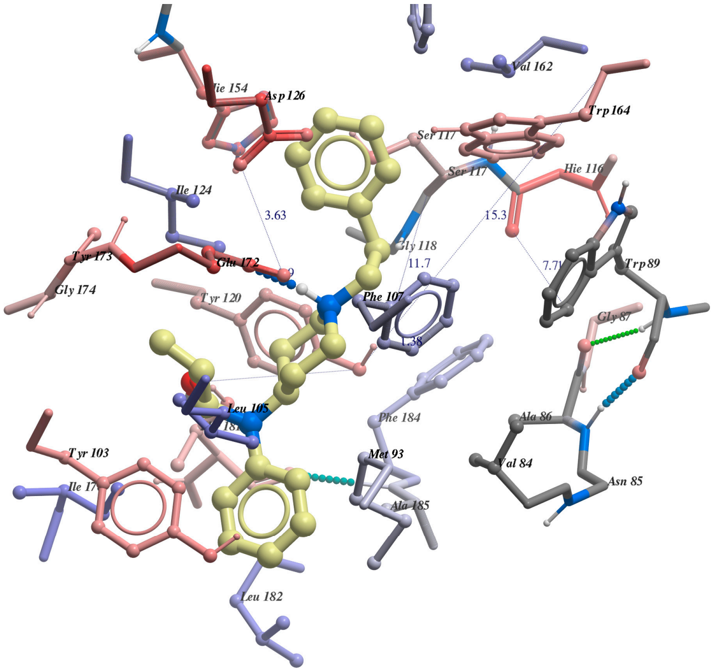

(b) HALO

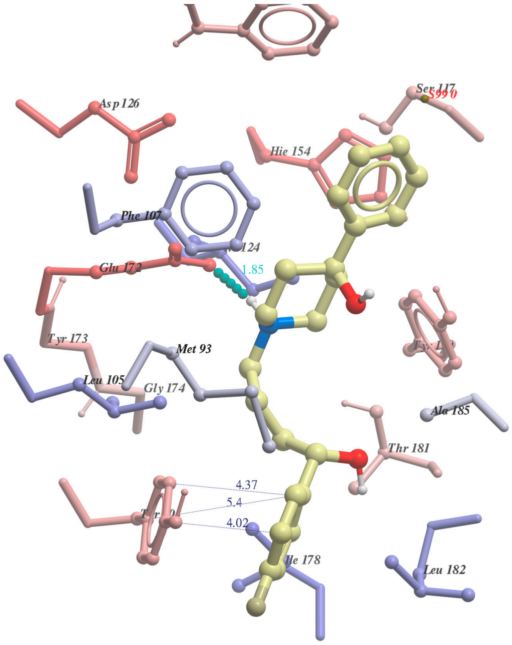

(c) MORPH

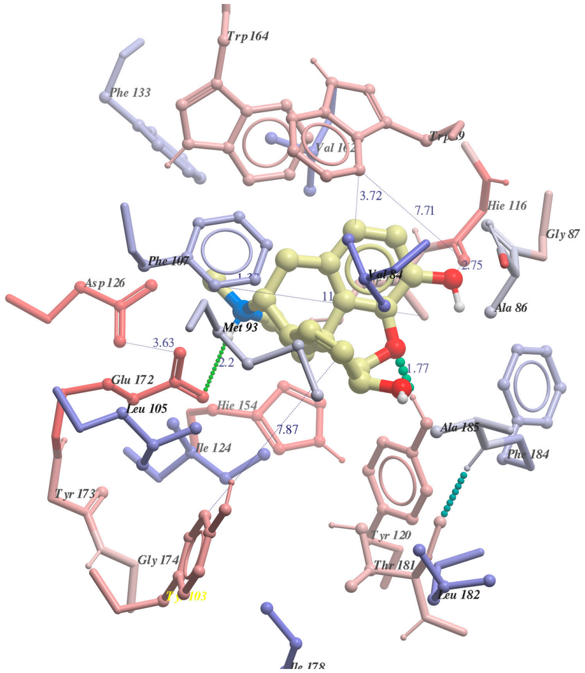
Trp 164
Phe 133
Val 162
Trp 89
3.72
7.71
Hie 116
Gly 87
Phe 107
Val 84
2.75
Asp 126
Ala 86
Met 93
3.63
1.77
2.2
Glu 172
Leu 105
Hie 154
Ala 185
7.87
Ile 124
Phe 184
Tyr 173
Tyr 120
Gly 174
Thr 181
Leu 182

(d) *S*-PTZ

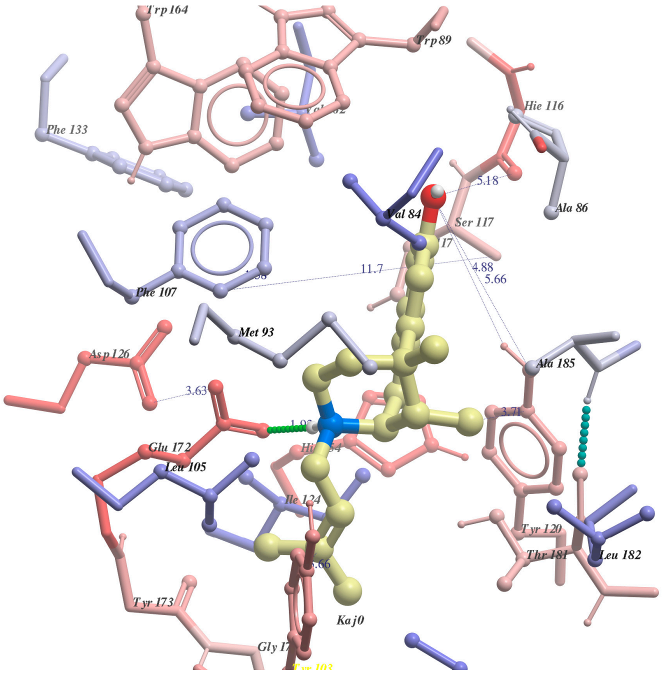
Trp 164
Trp 89
Hie 116
Phe 133
5.18
Ala 86
Val 84
Ser 117
4.88
11.7
5.66
Phe 107
Met 93
Ala 185
Asp 126
3.63
3.71
Glu 172
Leu 105
Ile 124
Tyr 120
Thr 181
Leu 182
Tyr 173
Kaj0

(e) *R*-PTZ

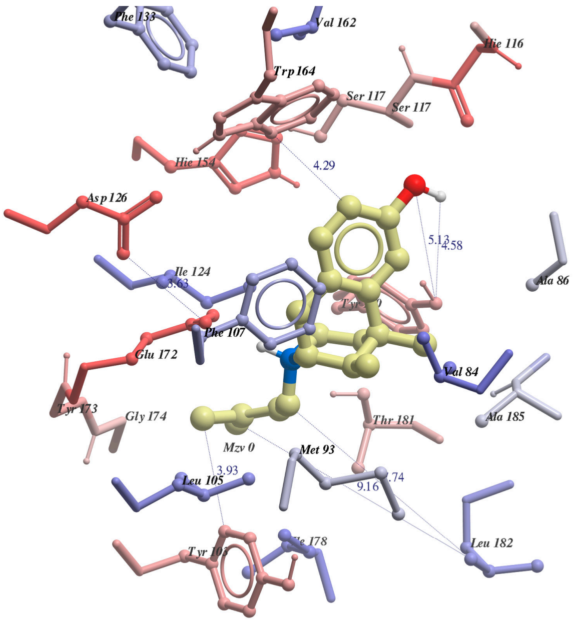

(f) *R*-PHZ

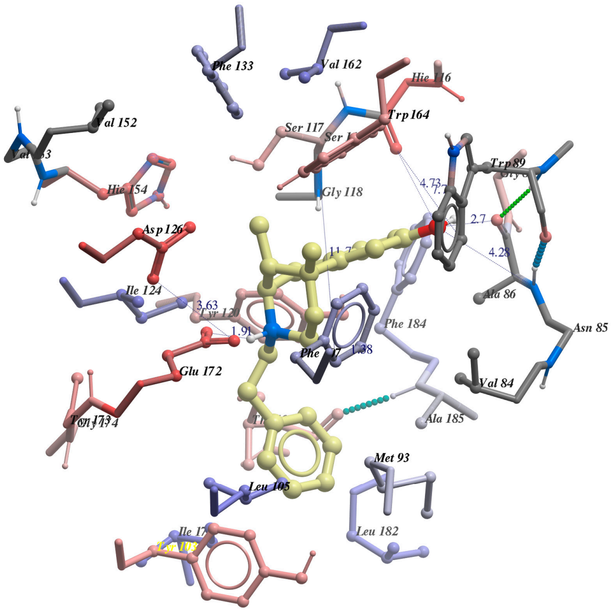

(g) *S*-PHZ

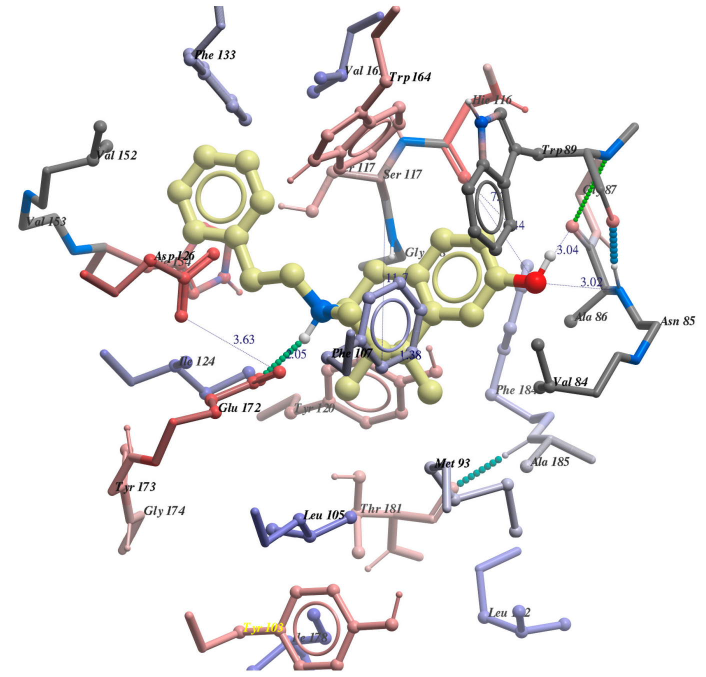
Phe 133
Trp 164
His 116
Val 152
Ser 117
Trp 89
Val 153
Asp 126
3.04
3.02
Ala 86
Asn 85
3.63
Ile 124
Phe 107
Glu 172
Tyr 120
Val 84
Phe 184
Met 93
Ala 185
Tyr 173
Gly 174
Leu 105
Thr 181

Table S1. The contacts between residues in receptor-ligand detected in the final MD snapshots. The names of different regions of the ligands: Ar1, Ar2, Ar(T), chair, Link, Me, -CH_2, see Fig.3.

Ligand	salt bridge	Hb between receptors residue-ligand	hydrophobic	CH- π
FENT	2.9	(Thr181) -(C=O): 3.05 Å	Ile178, Leu182, Ala185, Met93 with Ar2;<br>Leu105, Phe107 with chair<br>Phe133, Val162, Val152 with Ar1	Phe107- chair<br>Tyr103-Ar2<br>Phe133- Ar1<br>Tyr120-chair<br>Trp164-Ar1
HALO	2.8	COO^- (Thr181) (C=O): 3.7 Å<br>(Tyr120) (C=O):3.8 Å<br>Halogen bond: oxygen form OH group of Ser205 with Ar1 (C-F): 4.12 Å<br>oxygen form C=O group of Thr202 with Ar1 (C-F): 4.01 Å	Trp89, Trp164, Phe107 with Ar2;<br>Phe107, Tyr120 with chair<br>Thr181, Leu105, Tyr103, Tyr120 with link<br>Tyr103, Leu182 with Ar1	Trp89- Ar2<br>Tyr103- Ar1<br>Tyr120-chair, Ar2<br>Trp164-Ar2
MORPH	3.2<br>3.6	Hb oxygen atom OH (Tyr120) with oxygen<br>–O- (C-O):2.7 Å<br>Hb oxygen atom NH (Gly118) with oxygen<br>–O- (C-O): 4.0 Å<br>Hb oxygen atom C=O(backbone of Ala86)<br>with oxygen OH (OH):2.7 Å<br>Hb oxygen atom NH(backbone of Ala185)<br>with oxygen OH (OH):3.5 Å + CH3(sidechain of Ala185) with OH (OH): 3.22 Å	Trp89, Trp164 with Ar(T);<br>Phe107, Trp164 with CH2<br>Ile124 with chair<br>Val84 with Link	Trp89<br>Phe107<br>Tyr103<br>Tyr120<br>Trp164<br>Phe184
R-PTZ	2.9		Trp164, Phe107 with Ar(T);<br>Ala185 with Me<br>Phe107 with CH2<br>Tyr120 with chair<br>Ile178, Tyr103, Ile124, Leu105, Glu172 with Link	Phe107,<br>Tyr120
S-PTZ	2.9	Hb oxygen atom C=O(backbone of Ala86)<br>with oxygen OH (OH):3.2 Å	Ala185 with Me<br>Ile124 with CH2<br>Phe107 with chair<br>Ile178, Tyr103, Leu105, Glu172 with Link	Phe107,<br>Tyr120
R-PHZ	3.8	Hb oxygen atom C=O(backbone of Ala86) with oxygen OH (OH(T)):2.7 Å	Ala86, Phe184 with Ar(T);<br>Phe107, Trp164 with Me<br>Tyr120 with CH2<br>Phe107 with chair<br>Ile124 with Link<br>Ile178, Tyr103, Ala185, Thr181, Leu182 with Ar	Trp89<br>Tyr103<br>Phe107,<br>Tyr120<br>Trp164
S-PHZ	3.0	Hb oxygen atom C=O(backbone of Ala86) with oxygen OH (OH(T)):3.0 Å<br>Hb oxygen atom C=O(backbone of Trp89) with oxygen OH (OH(T)):3.2 Å	Ala86, Trp89, Val84 with Ar(T);<br>Tyr120, Ile124, Ala185 with Me<br>Phe107 with chair<br>Ile124 with Link<br>Val152, Phe133, Val162, His154 with Ar	Phe107,<br>Tyr120

Figure S3. Spectrum of the frequency of the contacts between receptor residues and atomic centers of ligands.

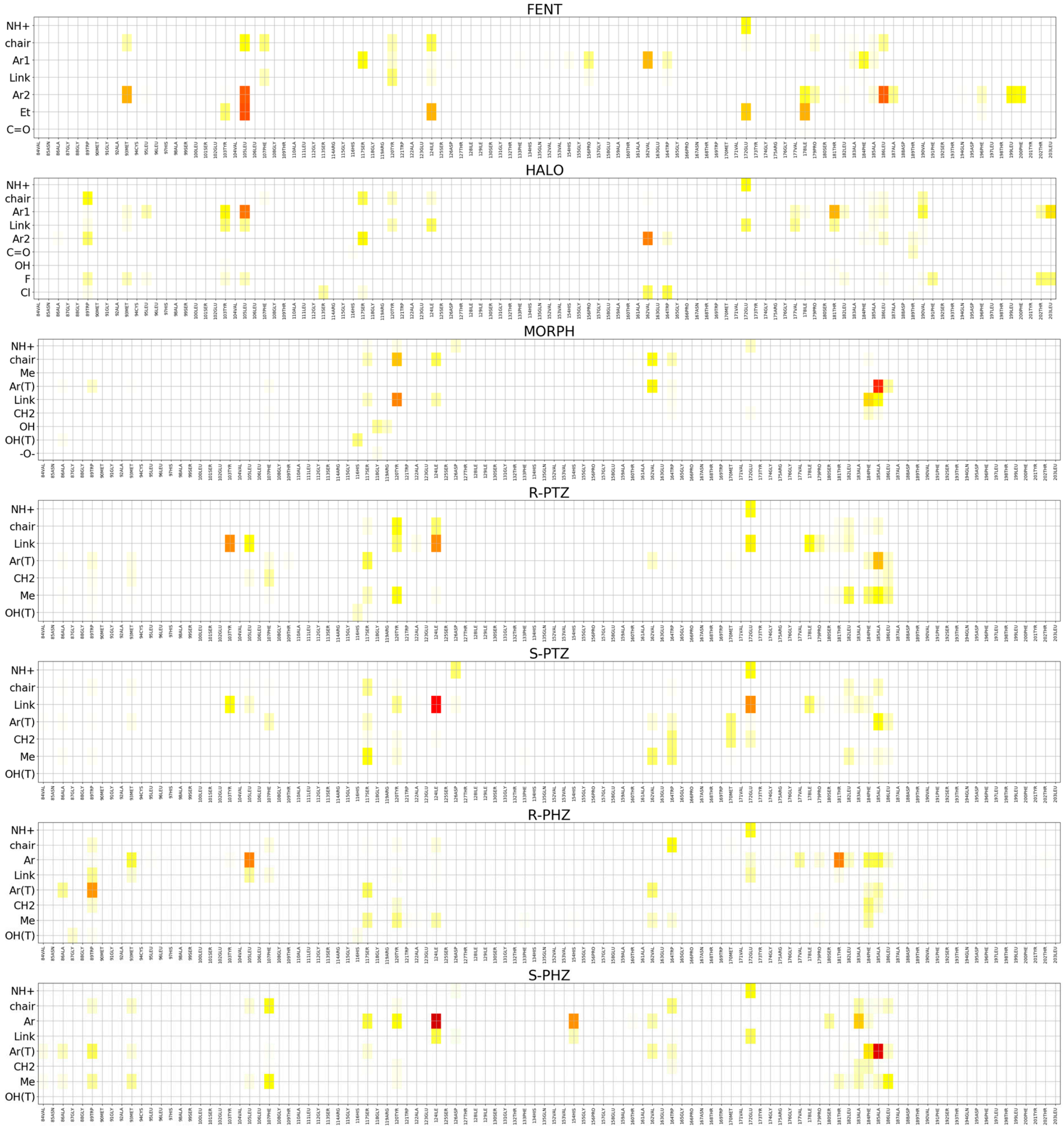

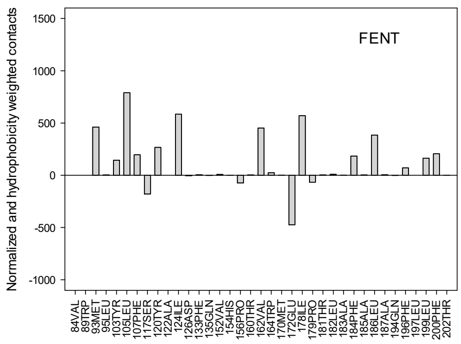

(a)

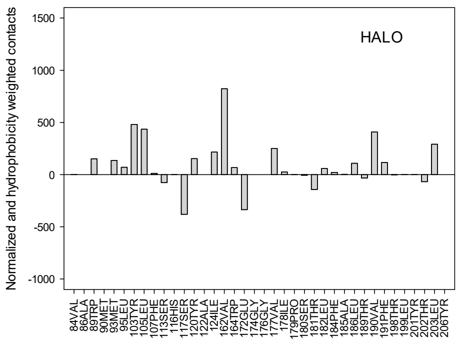

(b)

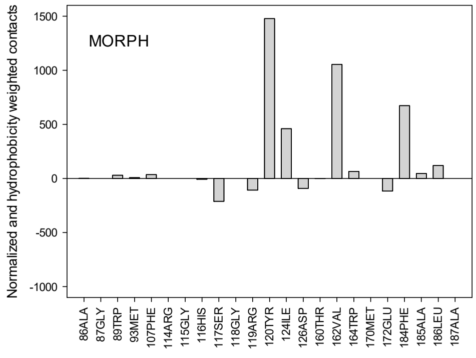

(c)

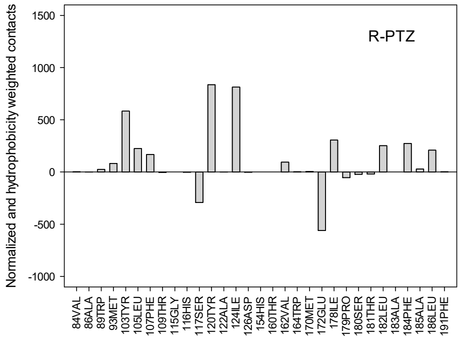

(d)

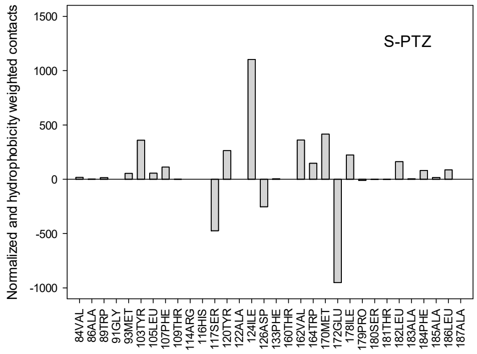

(e)

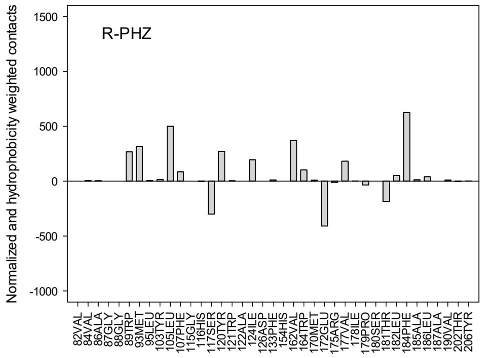

(f)

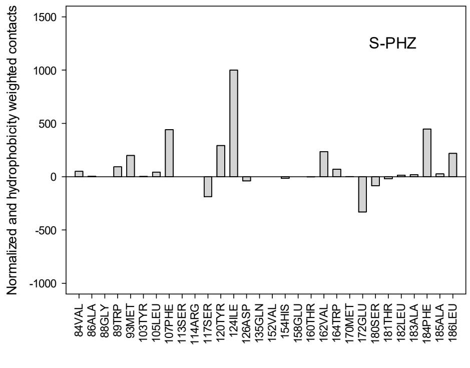

(g)

Figure S4, a-g. Spectrum of frequency of ligand contacts with receptor residues normalized according to the hydrophobic scale [40]. The positive bars correspond to hydrophobic while negative to hydrophilic contacts.
